# Molecular Characterization of Enterotoxigenic *Escherichia coli* Strains Isolated from Diarrheal Patients in Korea during 2003–2011

**DOI:** 10.1371/journal.pone.0096896

**Published:** 2014-05-19

**Authors:** Kyung-Hwan Oh, Dong Wook Kim, Su-Mi Jung, Seung-Hak Cho

**Affiliations:** 1 Division of Enteric Bacterial Infections, Center for Infectious Diseases, Korea National Institute of Health, Osong-eup, Chungcheongbuk-do, Republic of Korea; 2 Department of Pharmacy, College of Pharmacy, Hanyang University, Ansan, Kyeonggi-do, Republic of Korea; University of Malaya, Malaysia

## Abstract

Enterotoxigenic *Escherichia coli* (ETEC) is one of the major causes of infectious diarrhea in developing countries. In order to characterize the molecular features of human ETEC isolates from Korea, we investigated the profiles of enterotoxin and colonization factor (CF) genes by polymerase chain reaction (PCR) and performed multilocus sequence typing (MLST) with a total of 291 ETEC strains. The specimens comprised 258 domestic strains isolated from patients who had diarrhea and were from widely separated geographic regions in Korea and 33 inflow strains isolated from travelers visiting other Asian countries. Heat-stable toxin (STh)-possessing ETEC strains were more frequent than heat-labile toxin (LT)-possessing ETEC strains in the domestic isolates, while the detection rates of both enterotoxin genes were similar in the inflow isolates. The profile of CF genes of domestic isolates was similar to that of inflow isolates and the major CF types of the strains were CS3-CS21-CS1/PCF071 and CS2-CS3-CS21. Most of these 2 CF types were detected in ETEC strains that possess both *lt* and *sth* genes. The major MLSTST types of domestic isolates were ST171 and ST955. Moreover, the 2 major CF types were usually found concomitantly with the 2 major MLST STs, ST171 and ST955. In conclusion, our genotyping results may provide useful information for guiding the development of geographically specific vaccines against human ETEC isolates.

## Introduction

Enterotoxigenic *Escherichia coli* (ETEC) is a major cause of diarrhea and diarrheal deaths among young children and travelers in developing countries [1], [2]. The major virulence factors of diarrhea-causing ETEC strains are enterotoxins, that is, a heat-labile toxin (LT) and a heat-stable toxin (ST), that induce the watery diarrhea. The LT is an AB5 toxin with similarities to cholera toxin; it binds to ADP ribosylates the guanyl-nucleotied alpha regulatory binding protein of the adenylcyclase system thereby causing increased cyclic AMP levels. The ST is a small peptide molecule that activates guanylylcyclase, leading to the production of increased intracellular levels of cyclic GMP. The presence of the LT and/or ST leads to alterations in cellular signaling pathways that ultimately trigger increased chloride secretion and watery diarrhea [3], [4]. The LT toxin is encoded by *elt*AB, whereas the ST toxin is encoded by 2 different genes, *est*A and *st*1, which produce STh (originally isolated from ETEC in humans) and STp (originally from a pig isolate) [3]–[5]. Many ETEC strains also produce surface colonization factors (CFs), which mediate adherence to the small intestinal wall. To date, over 25 human ETEC CFs have been described and divided into 3 different families: (1) CFA/I-like group, including CFA/1, CS1, CS2, CS4, CS14, and CS17; (2) CS5-like group, including CS5, CS7, CS18, and CS20; and (3) another unique group, including CS3, CS6, and CS10 to CS12 [2], [6].

Targeting virulence factors such as toxins and CFs are the most effective approaches for ETEC vaccine development strategy [7], [8]. Human ETEC strains that originate from the same ETEC lineage may have inherited many of the same epidemiological and phenotypic traits. Therefore, defining these lineages could provide an additional basis on which to understand ETEC epidemiology and for identifying new vaccine antigens. Although several methods are available for performing lineage definition studies, multilocus sequence typing (MLST) is now widely adopted. MLST is a sequence-based typing system based on determination of short nucleotide sequences (approximately 500 bp) of 7 housekeeping genes and has recently become the method of choice for bacterial typing [9].

To identify ETEC lineages in the present study, we performed MLST and phylogenetic analyses on a collection of human ETEC strains isolated from diarrheal patients in Korea and travelers in different developing countries. We have reported the distribution of the genes that encode enterotoxins and their associated CF types, as well as the antibiotic susceptibility profile of ETEC isolates obtained from patients with diarrhea between 2003 and 2011 in Korea.

## Materials and Methods

### Identification of Bacterial Isolates as ETEC

A total of 291 human clinical isolates were collected between the years 2003 to 2011 through a routine surveillance system, which performed laboratory examination to isolate clinical specimens from stools of diarrheal patients in Korea and positively identified as ETEC. To avoid any bias in data analysis, information related to the patients was blinded during the analysis of the isolates. Among these 291 isolates, 258 strains were isolated from patients with diarrhea in widely separated geographic regions in Korea (domestic isolates) and 33 ETEC strains were isolated from travelers visiting other Asian countries (1 from Mongolia, 3 from Vietnam, 1 from Egypt, 1 from China, 2 from Indonesia, 5 from India, 3 from Cambodia, 6 from Thailand, 1 from Turkey, 9 from Philippines, and 1 from Hong Kong) between 2010 and 2011 (inflow isolates). Individual colonies from positive ETEC samples were analyzed using separate polymerase chain reaction (PCR) assays for the LT and STh genes. Bacteria were directly inoculated into 3 mL of Luria-Bertani broth for enrichment and incubated overnight at 37°C under shaking conditions. After incubation, the enriched broth culture was centrifuged at 13,000 rpm (Sorvall, Biofuge Pico, Germany) for 1 min, and the pellet was heated at 100°C for 10 min. After centrifugation of the lysate, 5 mL of the supernatant was used for PCR. The primers used for detection of the LT genes were LT-F (GTACTTCGATAGAGGAACTCAAATGAATAT) and LT-R (ATTCTGGGTCTCCTCATTACAAGTATC), and those used for detection of STh genes were STh-F (TTCGCTCAGGATGCTAAACCA) and STh-R (TTAATAGCACCCGGTACAAGCAGG).

### Detection of CF Genes

To detect CF genes, multiplex PCR assays were performed using the primers shown in Table 1. PCR assays were carried out in a 50 mL volume with 2U DNA Taq polymerase (Takara Ex Taq, Japan) in a thermal cycler (PTC-100; MJ Research, Watertown, MA, USA) under the following conditions: initial denaturation at 94°C for 5 min; 30 cycles each of 94°C for 1 min, 55°C for 1 min, 72°C for 1 min; and final cycle at 72°C for 5 min. The amplified PCR products were analyzed by gel electrophoresis in 2% agarose gels stained with ethidium bromide, visualized with ultraviolet illumination, and imaged with the Gel Doc 2000 documentation system (Bio-Rad, Hercules, CA, USA).

**Table 1:** Primer pairs of multiplex PCR for the detection of CFs.

Multiplex PCR group	SFs	Primer name	Sequence (5′ to 3′)	Product size (bp)	References
Group 1	CFAI	CFAI-F	TGAGTGCTTCWGCAGTAGAGA	204	20
		CFAI-R	CAGCAAGTTTAACAATTACTTTTTTAGT		
	CS6	CS6-F	GGAGTGGTAAATGCAGGAAACT	416	20
		CS6-R	GTACCAGACGAATATCCGCTATTA		
	CS4	CS4-F	TGAGTGCTTCWGCAGTAGAGA	300	20
		CS4-R	AAGTCACATCTGCGGTTGATAGAG		
	CS14	CS14-F	TGAGTGCTTCWGCAGTAGAGA	357	20
		CS14-R	TACTATTCGAAACACCTGCCG		
Group 2	CS3	CS3-F	GGTCTTTCACTGTCAGCTATGAGTT	136	20
		CS3-R	TAATGTTAAATTATCCTGAGGAGCC		
	CS5	CS5-F	GCGTGACACGTCAGCTAATATAAAC	235	20
		CS5-R	GGCATTCATATCAATAGAAATATGAGAC		
	CS7	CS7-F	TGCTCCCGTTACTAAAAATACG	418	20
		CS7-R	GGCATTCATATCAATAGAAATATGAGAC		
	CS15	CS15-F	ATGCGTAGTAAATTATCCATTCTT	364	20
		CS15-R	CTACTATGGGCGTCGTCAT		
	CS18	CS18-F	TTTGCTGCACTGCCTGCGAA	502	20
		CS18-R	TAACAGTACCAGCTTTAACCTGAC		
	CS12	CS12-F	TTACGTCTCTGATCATGGCTGTTA	562	20
		CS12-R	ATAGTCATTACTGCATTTGCATCAAC		
Group 3	CS2	CS2-F	TCTGCTCGTATCAATACCCAAGTT	140	20
		CS2-R	GTGCCAGCGAATGAAACCTCTAAA		
	CS17/19	CS17/19-F	ACTCTRTCGCATTAACCTATTCT	169	20
		CS17/19-R	GTCACTTTCATCGGAATTTGCGAG		
	CS8	CS8-F	TATGAGCCTKCTGGAAGTYATCAT	526	20
		CS8-R	TATTGTAGTATTATCAGTAGCAGCCA		
	CS21	CS21-F	TATGAGCCTKCTGGAAGTYATCAT	292	20
		CS21-R	GTTATTACGCACTTCGTCTGGT		
	CS1/PCF071R	CS1/PCF071R-F	ACTCTRTCGCATTAACCTATTCT	334	20
		CS1/PCF071R-R	CCCTGATATTGACCAGCTGTTAGT		
	CS22	CS22-F	ATGCGTAGTAAATTATCCATTCTT	442	20
		CS22-R	CATTTTTTTGGAAGGCTTTAATA		
Group 4	CS13	CS13-F	TTGATGTGATGGTTATCGCTT	212	20
		CS13-R	AAAATCCAGGGTGGCGATCTG		
	CS20	CS20-F	ATGATTATGCCCTTTTAACTATGG	413	20
		CS20-R	CAAGTTTTTGATCGCTTCCAATA		

### Multilocus Sequence Typing (MLST) Analysis

ETEC isolates were analyzed by MLST. The 7-gene (st7) MLST system of the EcMLST (www.Shigatox.net/mlst) was used for MLST. The MLST is based on the sequencing of internal fragments of the 7 housekeeping genes (*aspC*, *clpX*, *fadD*, *icdA*, *lysP*, *mdh*, and *uidA*). PCR products of the genes were amplified for each isolate using the primers whose sequences have been shown in Table 2. All PCR reactions were performed in 50-µL volumes using 10–100 ng of boiled bacterial DNA as the template. PCR products were purified using the QIAquick PCR Purification Kit (Qiagen, Hilden, Germany) and were sequenced in both directions using the same primer as that used for PCR. The MLST sequence type (ST) for each combination of alleles from sequences of 7 housekeeping genes was acquired on the EcMLST website and new allele numbers and STs were submitted to the EcMLST website.

**Table 2:** Primer epairs used for MLST.

Locus	Primer name	Sequence (5′ to 3′)	Product size (bp)
aspC	aspC-F4	GTTTCGTGCCGATGAACGTC	594
	aspC-R7	AAACCCTGGTAAGCGAAGTC	
clpX	clpX-F6	CTGGCGGTCGCGGTATACAA	672
	clpX-R1	GACAACCGGCAGACGACCAA	
fadD	fadD-F6	GCTGCCGCTGTATCACATTT	580
	fadD-R3	GCGCAGGAATCCTTCTTCAT	
icdA	icd-F2	CTGCGCCAGGAACTGGATCT	669
	icd-R2	ACCGTGGGTGGCTTCAAACA	
lysP	lysP-F1	CTTACGCCGTGAATTAAAGG	628
	lysP-R8	GGTTCCCTGGAAAGAGAAGC	
mdh	mdh-F3	GTCGATCTGAGCCATATCCCTAC	650
	mdh-R4	TACTGACCGTCGCCTTCAAC	
uidA	uidA-277F	CATTACGGCAAAGTGTGGGTCAAT	658
	uidA-277R	CCATCAGCACGTTATCGAATCCTT	

### Phylogenetic Analysis

The MLST sequences were aligned using the ClustalW method, and the phylogenetic trees were generated using the maximum likelihood (ML) method implemented in the PAUP* (Phylogenetic Analysis Using Parsimony, 4.0 b10), RAxML Blackbox webserver. ML topologies were evaluated by bootstrap analysis of 100 ML iterations, implemented in the RAxML web server. Phylogenetic networks of MLST sequences were constructed by the median-joining algorithm using Network 4.6.

### Antimicrobial Susceptibility

Antimicrobial susceptibility testing for *E. coli* isolates was determined with the VITEK 2 automated system using AST- N169 Card (bioMérieux, France) according to the guidelines of the Clinical and Laboratory Standards Institute (CLSI). The following antibiotics were tested: ampicillin, amoxicillin/clavalanic acid, ampicillin/sulbactam, cephalothin, cefotaxime, cefotetan, cefoxitin, cefazolin, ceftriaxone, imipenem, chloramphenicol, gentamicin, amikacin, nalidixic acid, ciprofloxacin, tetracycline, trimethoprim/sulfamethoxazole. *E. coli* ATCC 25922 was used for quality control.

### Statistical Analysis

GraphPad Prism version 6 was used for statistical analysis. For comparisons of two variables, chi-square test or Fisher exact test was used. A *P* value <0.05 was considered statistically significant.

## Results

### Profile of Enterotoxin and CF Genes in Domestic and Inflow Isolates

The 291 human ETEC strains represented 3 different enterotoxin profiles: ETEC-LT strains, ETEC-STh strains and ETEC-LT/STh strains. The profile of enterotoxin genes of domestic isolates was somewhat different from that of inflow isolates. In the domestic isolates, ETEC-LT/STh strains constituted 47.3% of the isolates, while ETEC-LT and ETEC STh accounted for only 16.3% and 36.4%. As shown in Table 3, a greater number of STh-possessing ETEC (ETEC-STh and ETEC-LT/STh) strains were detected than LT-possessing ETEC (ETEC-LT and ETEC-LT/STh) strains (83.7% vs. 63.6%) in the domestic ETEC cases. The detection rates of the 3 enterotoxin types were similar in the inflow isolates: ETEC-LT (36.4%, 12 of 33 strains), ETEC-STh (33.3%, 11 of 33 strains), and ETEC-LT/STh (30.3%, 10 of 33 strains). The frequency of ETEC-LT/STh strains was significantly higher in the domestic isolates than in the inflow isolates (*P* value <0.05).

**Table 3:** Prevalence of enterotoxins of ETEC isolates.

Enterotoxin types	No. of Domestic isolates (n = 258)	No. of Inflow isolates (n = 33)	*P* value^a^
LT	42 (16.3%)	12 (36.4%)	0.008
STh	94 (36.4%)	11 (33.3%)	0.443
LT/STh	122 (47.3%)	10 (30.3)	0.047

a Data represent results of the Fisher exact test comparing toxin types in domestic isolates and inflow isolates.

**Figure 1:**
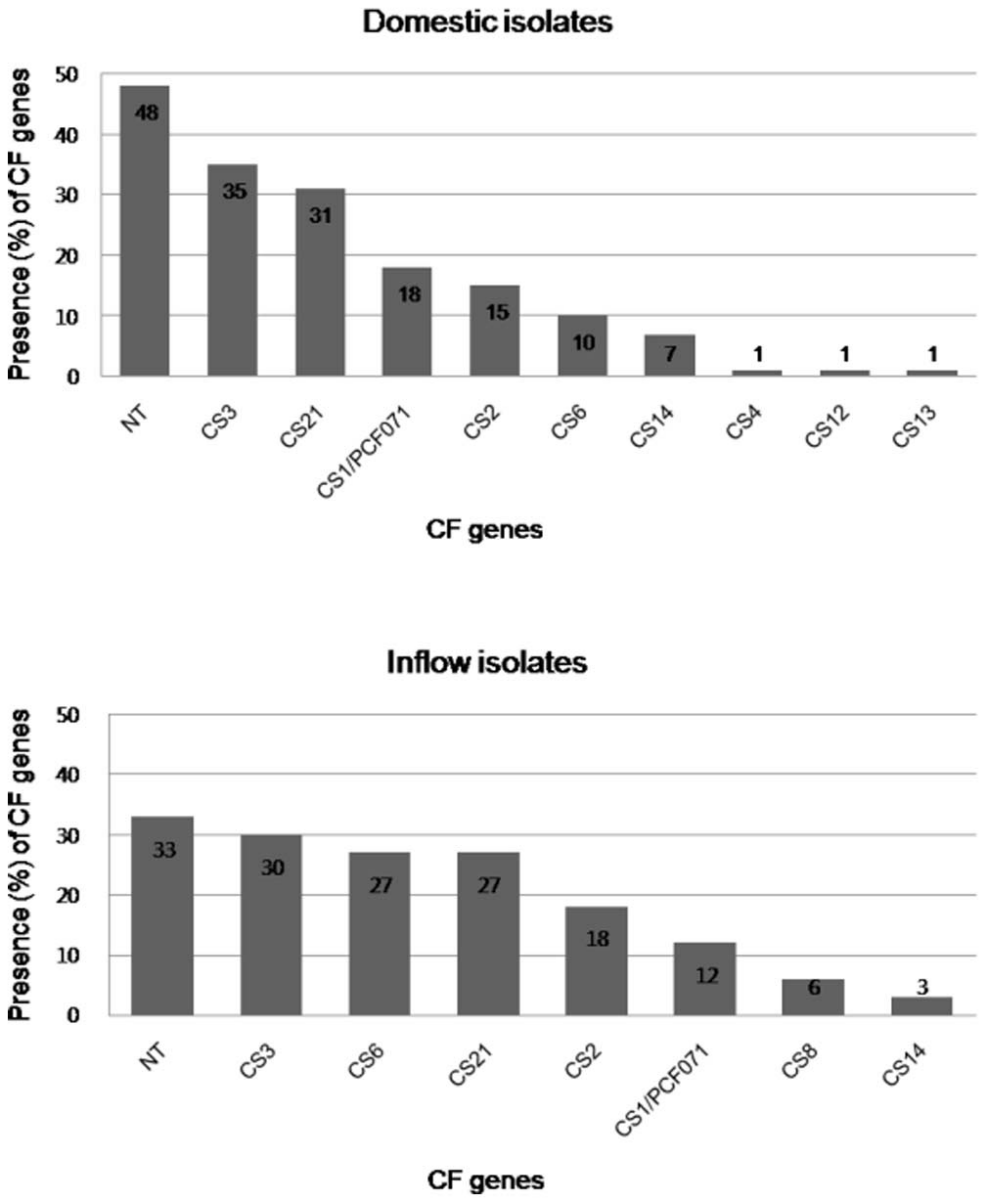
Profile of colonization factor (CF) genes in domestic and inflow isolates.

The CF gene profile of the domestic isolates was similar to that of inflow isolates. As shown in Figure 1, CS3 was predominantly isolated in both domestic and inflow human ETEC isolates (35% and 30%, respectively). In the domestic isolates, CS21, CS1/PCF071, CS2, CS6, and CS14 were frequently detected. In the inflow isolates, CS6, CS21, CS2, CS1/PCF071 and CS8 were frequently present. The proportion of CF-non typable strains was also high in both isolate groups (Figure 1 and Table S1, Table S2).

Ten non-typable strains were directly examined by electron microscopy. Seven (70%) among the ten strains possessed fimbriae (data not shown). This result indicates that the seven strains express CFs, but the genes encoding the CFs do not react with the PCR primers used in this study. Moreover, one hundred strains of all were re-confirmed CFs using mono-PCR with primers described in Rodas et al., 2009 (data not shown).

The prevalent types of CF genes in the domestic isolates were CS3-CS21-CS1/PCF071, CS2-CS3-CS21, CS6, and CS14 and the major CF types of the inflow isolates were CS3-CS21-CS1/PCF071, CS6, and CS2-CS3. Interestingly, most strains of the CS3-CS21-CS1/PCF071 and CS2-CS3-CS21 types were detected in ETEC-LT/STh strains. The CS6 type was found more often in LT-possessing ETEC strains, while the CS14 type was more frequent in STh-possessing ETEC strains (Table 4).

**Table 4:** Prevalence of CFs and enterotoxins of ETEC isolates.

CF types	Domestic isolates				Inflow isolates			
	No. of CFs-type	No. of LT	No. of STh	No. of LT/STh	No. of CFs-type	No. of LT	No. of STh	No. of LT/STh
CS3-CS21-CS1/PCF071	38	0	2	36	4	0	0	4
CS2-CS3-CS21	31	0	0	31	1	0	0	1
CS6	22	11	7	4	6	6	0	0
CS14	15	2	9	4	0	0	0	0
CS2-CS3	5	1	0	4	5	0	1	4
CS3	6	0	0	6	0	0	0	0
CS3-CS21	6	0	0	6	0	0	0	0
CS21-CS1/PCF071	3	0	0	3	0	0	0	0
CS21	1	0	0	1	3	0	3	0
CS3-CS1/PCF071	3	0	0	3	0	0	0	0
CS6-CS8	0	0	0	0	2	1	0	1
CS3-CS4-CS1/PCF071	1	0	0	1	0	0	0	0
CS2	2	0	1	1	0	0	0	0
CS12	1	1	0	0	0	0	0	0
CS13	1	1	0	0	0	0	0	0
CS4-CS6	1	1	0	0	0	0	0	0
CS4-CS6-CS21	0	0	0	0	1	0	1	0
NT	122	25	75	22	11	5	6	0
Total	258	42	94	122	33	12	11	10

NT; non typable.

### MLST Analyses of the Isolates

**Figure 2:**
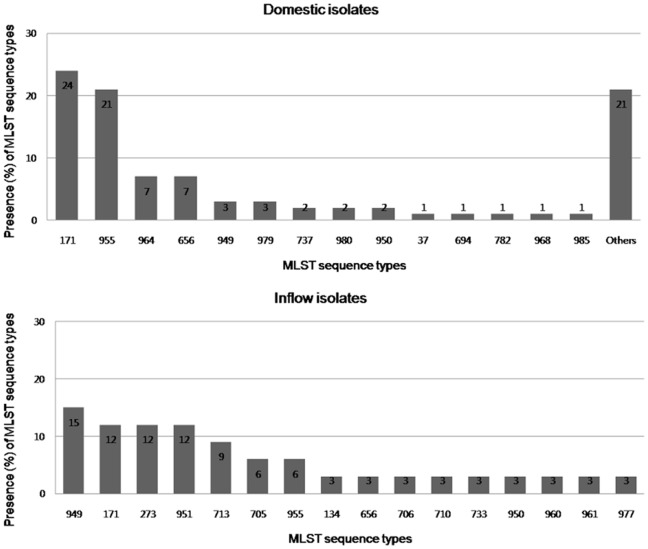
MLST sequence types in domestic and inflow isolates.

MLST of the 258 domestic human ETEC isolates represented 65 different MLST STs: 21 sequence types were known STs, and 44 new STs were recovered (Table S3). ST171 were the most common, with a frequency of 24% (62/258) and were constantly represented in each year between 2003 and 2011. The other predominant STs were ST955 (21%, 53/258) and ST964, ST656 (7%, 18/258) (Figure 2 and Table S1). Analysis of the 33 inflow isolates identified 16 different STs, which contained 7 new STs (43.8%, Table S3). The most frequent ST type was ST949, that is, 15% (5/33), and this was followed by ST171, ST273, ST951 (12%, 4/33), ST713 (9%, 3/33) and ST705, ST955 (6%, 2/33) (Figure 2 and Table S2). Interestingly, 8 STs (ST273, ST706, ST710, ST713, ST733, ST951, ST960, and ST961) were found only in inflow ETEC isolates, and not in domestic isolates. ST171 strains represented various CF types, including 2 major CF types (CS3-CS21-CS1/PCF071 and CS2-CS3-CS21) and other CF types (CS6, CS3, CS21-CS1/PCF071, CS2-CS3, CS3-CS1/PCF071, CS14, and CS12). ST955 strains also showed various CF types: CS3-CS21-CS1/PCF071, CS2-CS3-CS21, CS3-CS21, CS3-CS1/PCF071, CS2-CS3, CS6, and CS12. As shown in Table 5, 2 major CF types, CS3-CS21-CS1/PCF071 and CS2-CS3-CS21, were usually found in the 2 major domestic MLST STs, ST171 and ST955; 89% (34/38) of strains possessing the CS3-CS21-CS1/PCF071 type and 93% (29/31) of strains possessing the CS2-CS3-CS21 type were found in the 2 major MLST STs. However, there were more NT (not typeable) CF types in the strains of other MLST STs.

**Table 5:** Prevalence of MLST sequence types and CF types of domestic and inflow isolates.

Isolate groups	Major MLST sequence types (n)	No. of major – and oter CF types						
		CS3-CS21-CS1/PCF071	CS2-CS3-CS21	CS6	CS14	CS2-CS3	Etc.	NT
Domestic isolates	ST171 (62)	15	13	3	0	2	8	21
	ST955 (53)	19	16	1	0	2	7	8
	ST964 (18)	0	1	0	0	0	0	17
	ST656 (18)	0	0	1	0	0	0	17
	ST949 (8)	2	0	2	0	0	0	4
Inflow isolates	ST949 (5)	0	0	1	0	0	1	3
	ST171 (4)	0	1	0	0	0	0	3
	ST273 (4)	4	0	0	0	0	0	0
	ST951 (4)	0	0	0	0	4	0	0

etc.: Includes types present at lower incidence than the major types, NT; non typable.

**Figure 3:**
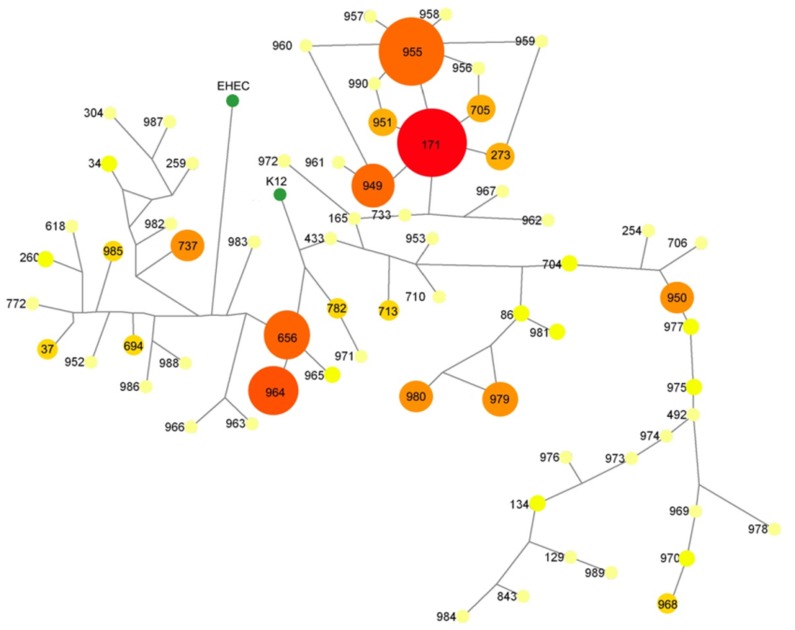
Phylogenetic networks of the isolates according to MLST STs. The sizes of the circles are proportional to the number of MLST STs. The major MLST STs such as ST171 and ST955 were 23% (66/291) and 19% (55/291).

The phylogenetic trees of the STs analyzed above were generated and compared with those of 3 other reference strains (*E. coli* K12, EHEC EDL933, and ETEC H10407). Phylogenetic analysis showed that 7 STs (ST971, ST782, ST965, ST656, ST964, ST966, and ST963) were closely related to those of *E. coli* K12. EHEC EDL933 also has a close phylogenetic relationship with some STs such as ST988, ST737, ST985, ST986, ST982, and ST259. ETEC H10407 represented the ST171 type as similar as other many ETEC strains (Figure 3).

### Antibiotic Resistance Analyses of the Isolates

Antibiotic resistance rates of domestic and inflow isolates are shown in Table 6. The isolates of both groups showed a higher resistance to ampicillin (30% and 49%, respectively), nalidixic acid (38% and 36%, respectively), and trimethoprim-sulfamethoxazole (26% and 27%, respectively) when compared with other antibiotics. Resistance to imipenem and amikacin was not found in any ETEC strains. The inflow isolates conferred a stronger resistance to antibiotics of the cephem class. However, no significant association was found between the ETEC genotype and antimicrobial susceptibility.

**Table 6:** Comparison of antibiotic resistance of domestic and inflow isolates.

Antibicrobial agents	Antibiotic resistances (%) of isolates	
	Domestic isolates	Inflow isolates
**β-lactams**		
Ampicillin (AM)	30	49
**β-lactam/β-lactamase inhibitor combinations**		
Ampicillin-sulbactam (SAM)	11	28
Amoxicillin/Clavulanic Acid (AMC)	3	3
**Cephems**		
Cephalothin (CF)	12	27
Cefazolin (CZ)	10	21
Ceftriaxone (CRO)	3	12
Cefotaxime (CTX)	3	12
Cefoxitin (FOX)	2	0
**Carbapenems**		
Imipenem (IPM)	0	0
**Quinolones**		
Nalidixic Acid (NA)	38	36
Ciprofloxacin (CIP)	2	0
**Aminoglycosides**		
Amikacin (AN)	0	0
Gentamicin (GM)	5	0
**Tetracyclines**		
Tetracycline (TE)	29	18
**Phenicols**		
Chloramphenicol (C)	3	0
**Folate pathway inhibitors**		
Trimethoprim-sulfamethoxazole (SXT)	26	27

## Discussion

ETEC is an important pathogen responsible for traveler’s diarrhea and causes about 700,000 childhood deaths per year, mostly in young children [10]. A variety of strategies have been pursued in attempts to develop a vaccine against ETEC. The most promising vaccine candidate to date is a killed whole-cell vaccine comprising different ETEC strains that express the most prevalent enterotoxins and CFs [11], [12].

The relative proportions of enterotoxin- and CF-possessing isolates seem to vary from 1 geographical area to another in both diarrhea and control patients with ETEC infection [2]. Our results also showed that the proportions of enterotoxins were different between the domestic and inflow isolates. The proportion of enterotoxin types of the domestic isolates identified here is similar to those for other studies conducted in Bangladesh [13] and Egypt [14], while other studies in Argentina [15], India [16], and Peru [17] reported that LT-producing ETEC were predominant. Clemens et al. [19] reported that ETEC strains that only express the LT are considered less important as pathogens. The identification of CFs is more important for epidemiological studies and for the development of CF-based vaccines against ETEC because ETEC organisms carrying more than 1 CF gene are common [20], [21]. In this study, CS3 and CS21 were the most prevalent CFs detected in both domestic and inflow strains. In other studies, CFA/I was the most prevalent CF type [2], [14], [18], while strains possessing CFA/1 were rarely detected in this study. Interestingly, CS6 was found more in inflow strains (17%) than domestic strains (6%). Thus, variations in the prevalence of CF antigens may be related to location. Of strong interest, 2 major prevalent CF gene types were found in ETEC strains isolated in Korea. Moreover, we observed a strong correlation between some CF genes and enterotoxin types. In brief, we have demonstrated that STh and CS3/CS21 genes are the most prevalent enterotoxin and CF genes in the domestic ETEC isolates in Korea.

Resistance to common antibiotics such as ampicillin, tetracycline and trimethoprim-sulfamethoxazole was frequently detected in ETEC strains, consistent with reports from other authors [22]–[27]. High-level resistance to antibiotics has developed in part because of heavy clinical use of antibiotics, since these drugs are associated with excellent safety and have a low cost. Therefore, empirical use of these antibiotics should be limited. Moreover, among the cephems, high resistance to cephalothin underscores the importance of ongoing surveillance. However, we did not find any association between the genotypes and antibiotic susceptibility profiles of ETEC strains.

MLST is a very useful tool for determination of bacterial lineages [28]–[30]. Therefore, we used MLST to characterize ETEC lineages in association with the distribution of enterotoxin and CF genes. In general, we observed that different MLST STs were associated with different CFs. These genes appeared to be spread across several different lineages. Such genes have been reported to spread through horizontal transfer by transposable elements [31], [32]. However, a strong association was found between the major CF types and MLST types. Two major CF types were usually found in 2 major MLST STs, that is, ST171 and ST955, in the domestic isolates.

Currently, there is no licensed vaccine for use against ETEC diarrhea. To our knowledge, this study is the first to report the distribution of enterotoxin and CF genes in ETEC strains from Korea. We have also provided information regarding ETEC lineages, obtained using MLST, and the antimicrobial susceptibility profile of the isolates. Our data on the frequency and geographic association of the various antigenic virulence factors will provide useful information for strategies to develop novel and effective anti-ETEC vaccines.

## Supporting Information

Table S1 **Profile of enterotoxin- and CF genes, MLST sequence types and antobiotic resistance of domestic ETEC isolates.**
(XLSX)

Table S2 **Profile of enterotoxin- and CF genes, MLST sequence types and antobiotic resistance of inflow ETEC isolates.**
(XLSX)

Table S3 **New MLST sequence types of domestic and inflow ETEC isolates.**
(XLSX)
